# The recurrence and mortality risk in Luminal A breast cancer patients who lived in high pollution area

**DOI:** 10.1371/journal.pone.0335140

**Published:** 2025-10-17

**Authors:** Pimwarat Srikummoon, Patrinee Traisathit, Wimrak Onchan, Chagkrit Ditsatham, Natthapat Thongsak, Nawapon Nakharutai, Salinee Thumronglaohapun, Titaporn Supasri, Phonpat Hemwan, Imjai Chitapanarux

**Affiliations:** 1 Department of Statistics, Faculty of Science, Chiang Mai University, Chiang Mai, Thailand; 2 Division of Radiation Oncology, Department of Radiology, Faculty of Medicine, Chiang Mai University, Chiang Mai, Thailand; 3 Head Neck and Breast Unit, Department of Surgery, Faculty of Medicine, Chiang Mai University, Chiang Mai, Thailand; 4 Atmospheric Research Unit of National Astronomical Research Institute of Thailand, Chiang Mai, Thailand; 5 Department of Geography, Faculty of Social Sciences, Chiang Mai University, Chiang Mai, Thailand; Menzies School of Health Research: Charles Darwin University, AUSTRALIA

## Abstract

Luminal A is the most common subtype of breast cancer and has the best prognosis comparing to the others. The association between air pollution and survival of breast cancer have been reported but not specific to this subtype. We examined pollutant distributions over a decade in upper Northern Thailand, the area of high average annual particulate matter levels, and their impact on the mortality and recurrence risks of patients with luminal A breast cancer. Retrospective data of 1,305 luminal A breast cancer patients diagnosed from 2003 to 2018 were enrolled to this study. Cox proportional hazard models were used to identify factors associated with mortality and recurrence risks including all known risk factors and the annually averaged concentrations of pollutants. On multivariable analysis; metastatic stage (adjusted hazard ratio (aHR) =10.50; 95% confidence interval (95%CI): 7.23–15.25), smoking history (aHR = 1.72; 95% CI: 1.14–2.60), and age ≥ 50 years old (aHR = 1.46; 95% CI: 1.13–1.90) were significant factors influencing mortality risk. Factors contributing to recurrence risk included metastatic stage (aHR) 4.96 (95% CI: 2.78–8.83) and exposure to the time-updated local concentration of PM_10_ > 55 µg/m^3^ (aHR = 1.68; 95% CI: 1.16–2.45). Exposure to air pollutants is one of the detrimental factors affected to recurrence and mortality in luminal A subtype breast cancer.

## Introduction

The luminal A breast cancer subtype comprises a substantial proportion (50–60%) of the collective occurrence of breast cancers [1]. Within the specific context of Thailand, luminal A breast cancer has been observed in 46% of reported cases [2]. The identification of this subtype is predicated upon its characteristic estrogen receptor-positive (ER+), progesterone receptor-positive (PR+), and human epidermal growth factor receptor 2-negative (HER2–) statuses [1,3]. It is noteworthy that luminal A breast cancer is associated with a favorable prognosis and exhibits a notable degree of responsiveness to hormone therapy [4,5].

Based on the existing literature, it has consistently been reported that individuals diagnosed with luminal A breast cancer demonstrate a reduced risk of recurrence [6,7] and an increased overall survival (OS) rate compared to those with other subtypes [7–10]. Noteworthy factors associated with an elevated risk of recurrence of luminal A breast cancer include advancing age [11], alcohol consumption [12], smoking habit [13], high body mass index (BMI) [14,15], and the specific stage of cancer progression [12,16].

Exposure to air pollutants, notably nitrogen dioxide (NO_2_), particulate matter (PM) (PM_2.5_ (PM ≤ 2.5 µm) and PM_10_ (PM ≤ 10 µm)), has been positively correlated with an elevated risk of mortality in individuals diagnosed with breast cancer [17–21]. Nevertheless, there is a conspicuous gap in the literature concerning investigation of the connection between air pollution and mortality risk of breast cancer patients with the luminal A subtype [22]. In addition, the potential connection between air pollution and the recurrence risk in luminal A breast cancer cases has not been adequately explored. Consequently, it is imperative to thoroughly examine the effect of air pollution on both the survival and recurrence probabilities of individuals affected by luminal A breast cancer.

The levels of PM_2.5_, PM_10_, nitrogen dioxide (NO_2_), sulfur dioxide (SO_2_), carbon monoxide (CO), and ozone (O_3_) in the upper northern region of Thailand spanning Chiang Mai, Chiang Rai, Nan, Phrae, Phayao, Lampang, Lamphun, and Mae Hong Son provinces consistently exceed the guidelines established by the World Health Organization (WHO) [23]. Especially, Chiang Mai province has been acknowledged as one of the world’s most hazardous air pollution locations, particularly during the dry season (January–April) [24,25]. The heightened pollution in this region is principally ascribed to various contributors, including smoke emanating from forest fires and biomass burning [26,27], cross-border pollution [28], and pronounced transportation activity [29]. The upper northern region of Thailand is characterized by a multitude of mountain ranges that play a pivotal role in the accumulation of air pollution in the area [30,31]. Consequently, the authorities are persistently grappling with severe air pollution from PM, which has constituted an enduring issue for over a decade [31].

In this study, we primarily investigated whether exposure to air pollutants PM_2.5_, PM_10_, NO_2_, SO_2_, CO, and O_3_ over the past decade has influenced the survival and recurrence rates of luminal A breast cancer by using time-varying covariates within a Cox proportional hazard model. In addition, we also assessed the risk of each 10-unit increase in annual concentrations of PM_2.5_, PM_10_, CO, and O_3_ and each 1-unit increase in annual concentrations of NO_2_ and SO_2_ among luminal A breast cancer patients by utilizing Poisson regression models (types of generalized linear models (GLM)) and distributed lag models.

## Materials and methods

### Study population

Women diagnosed with luminal A breast cancer between January 1, 2003, and December 31, 2018, were identified from the Chiang Mai Cancer Registry located at Chiang Mai University Hospital. This registry is the primary repository for cancer statistics for the upper northern region of Thailand covering Chiang Mai, Chiang Rai, Nan, Phrae, Phayao, Lampang, Lamphun, and Mae Hong Son provinces. Our methodology involved a systematic review of the pathological diagnostic data, from which we categorized breast cancer subtypes based on the expression statuses of ER, PR, and HER2. ER and PR are considered to be positively expressed when present in at least 1% of the cell nuclei while HER2 is considered to be positively expressed when at least 1% of cells showed uniform, intense, and complete membrane staining for it [32,33]. Subsequently, cohort members were grouped according to the classification of luminal A breast cancer as ER + , PR + , and HER2– [3,34]. We access this clinical data on May 25, 2021.

### Data collection and measurements

Demographic data on the patients encompassing the age at the time of diagnosis, BMI, histories of smoking and alcohol use, and precise cancer staging based on the Surveillance, Epidemiology, and End Results (SEER) classification system, which delineates stages as localized, regional, or metastatic, were used in the analysis. Since the tumor grade, histology, and treatment for each luminal A breast cancer patient were not systematically linked between hospital medical records and the Chiang Mai Cancer Registry, these variables that influenced recurrence and survival were not included in this study.

The follow-up duration is from the moment of diagnosis to the occurrence of death irrespective of the cause or until the last recorded follow-up date. In addition, instances where data were lost due to censoring were accounted for by employing the termination of the study period (December 31, 2020) as a reference point and contingent upon which event transpired first. This rigorous methodology ensured a comprehensive analysis of patient outcomes within the specified timeframe.

Hourly concentrations of PM_2.5_, PM_10_, NO_2_, SO_2_, CO, and O_3_ were obtained from the Copernicus Atmosphere Monitoring Service (CAMS) and the European Centre for Medium-Range Weather Forecasts [35–37]. The CAMS reanalysis datasets derived from global observational data assimilated through a modeling system provide atmospheric composition data at an approximate spatial resolution of 80 kilometers. These data are available as either spectral coefficients with T255 triangular truncation (approximately 80 km) or a reduced N128 Gaussian grid. The CAMS datasets (generated using a physics- and chemistry-based atmospheric model [38,39]) are appropriate for climatological calculations, trend analyses, and comparative studies with other reanalysis models [37]. The data’s reliability and validity (documented on the CAMS Quality Assurance website) indicate the integrated system’s utility for estimating observational bias and differentiating high-quality data from inaccurate data. The atmospheric model generates estimates for areas with limited data or for atmospheric constituents for which direct observations are unavailable. Reanalysis offers significant advantages in ensuring spatiotemporal data completeness at each global grid point over extended periods and in a uniform format [40]. We accessed the three-hour ambient air pollution concentration levels of PM_2.5_ (kg/m^3^), PM_10_ (kg/m^3^), NO_2_ (kg/m^2^), SO_2_ (kg/m^2^), CO (kg/m^2^), and O_3_ (kg/m^2^) in upper northern Thailand for the study period (accessed on 27 November 2021). The concentration of each pollutant was calculated from the annual average concentrations for each district separately in the eight provinces in upper northern Thailand. Subsequently, the annual average concentrations were linked to the district of each patient’s residence based on the assumption that the district recorded in the patient’s information corresponded to their actual residence.

### Ethical approval

Ethical approval was granted by the Chiang Mai University Ethics Committee (No 200/2021) in the Faculty of Medicine. Due to anonymous data recorded in the present study, the requirements for written informed consent were waived by the Research Ethics Committee of the Faculty of Medicine at Chiang Mai University.

### Statistical analysis

The characteristics of patients are presented using medians and interquartile ranges (IQRs) for continuous data, while categorical data are portrayed as frequencies and percentages. Moreover, the annually averaged concentrations of PM_2.5_, PM_10_, NO_2_, SO_2_, CO, and O_3_ were segmented into two categories using quartiles, to which dichotomization was applied when deemed suitable. The age at diagnosis was categorized as < 50 and ≥50 years old and BMI categorized was as <30 and ≥30 kg/m^2^. These were adhered to due to the recommended cut-off point [41,42].

The overall rate of death and the rates for each variable were calculated as the number of deaths divided by the total number of person-years of follow-up (PYFU). The overall rate of recurrence was calculated as the number of recurrences divided by the total number of PYFU. Confidence intervals (CIs) for the mortality rates and recurrence rates were obtained from Poisson distributions. OS rates and recurrence-free survival (RFS) rates were created by using Kaplan–Meier curves, and log-rank tests were used to test for significant differences between the survival probabilities of the groups for each variable. Cox proportional hazard models were used to identify factors associated with mortality and cancer recurrence risks. Those with a *p*-value<0.25 in the univariate analysis were included in the multivariate analysis via a backward elimination procedure, except for variables with a lot of missing values or high correlation (multicollinearity), or else where the Kaplan–Meier curve intersected after the period of the study.

We also assessed the risk of each 10-unit increase in annual concentrations of PM_2.5_, PM_10_, CO, and O_3_ and each 1-unit increase in annual concentrations of NO_2_ and SO_2_ among luminal A breast cancer patients by utilizing Poisson regression models and distributed lag models. The selection of different unit increments is based on the variability of the annual average concentration distributions. NO_2_ and SO_2_ show relatively low concentration variability annually, thus necessitating a 1-unit increment for exposure consideration. Conversely, PM_2.5_, PM_10_, and CO demonstrate higher variability in annual average concentrations, thus requiring a 10-unit increment for exposure assessment (S1 Fig). To control time-varying confounders, models were adjusted for long-term trends and seasonality. Both constrained and unconstrained distributed lag models were tested to determine the most appropriate effect patterns. The effect estimates are presented as the relative risk (RR) with 95% CI, representing the change in risk per 1-unit increase in annual concentrations of NO_2_ and SO_2_ and per 10-unit increase in annual concentrations of PM_2.5_, PM_10_, CO, and O_3_. To determine the optimal lag length for the model, we considered the Likelihood Ratio (LR) test to assess whether additional lags significantly improve model fit and evaluated the Final Prediction Error (FPE), Akaike Information Criterion (AIC), Hannan-Quinn Information Criterion (HQ), and Schwarz Bayesian Information Criterion (SBIC), with lower values indicating better model performance. All the analyses were performed by using STATA (version 12).

## Results

The cohort used in the study comprised 1,305 patients who received a diagnosis of luminal A breast cancer between January 2003 and December 2018. The demographic profile of the patients revealed a median age of 52 years old at diagnosis (IQR: 45–60) and a median BMI of 23.9 kg/m^2^ (IQR: 21.5–26.4). The distribution of cancer stages exhibited 63% for localized cases, 30% for regional cases, and 5% for metastatic instances. Further exploration of the patients’ histories revealed that 5% of the cohort had a history of smoking while 8% had a history of alcohol use. These delineated statistics provide a comprehensive overview of the demographic and clinical characteristics within the specified timeframe.

### Baseline characteristics and mortality rate

In Table 1, 288 patients afflicted with luminal A breast cancer had a mortality rate of 3.4 per 100 PYFU (95%CI: 3.1–3.9). Specifically, the mortality rate for older patients reached 3.9 per 100 PYFU (95%CI: 3.4–4.5), while those with a low BMI exhibited a mortality rate of 3.6 per 100 PYFU (95%CI: 2.2–5.8).

**Table 1:** Baseline characteristics and the associated mortality rate of the study population.

Characteristic	Survival (n (%))	Mortality (n (%))	PYFU	Mortality Rate	95%CI
Overall	1,017 (78%)	288 (22%)	8369	3.4	3.1–3.9
Age at diagnosis (years old)					
< 50	426 (80%)	104 (20%)	3687	2.8	2.3–3.4
≥ 50	591 (76%)	184 (24%)	4682	3.9	3.4–4.5
BMI (kg/m^2^)/416					
< 30	674 (83%)	140 (17%)	5367	2.6	2.2–3.1
≥ 30	58 (77%)	17 (23%)	473	3.6	2.2–5.8
Cancer stage/22					
Local	683 (83%)	137 (17%)	5843	2.3	2.0–2.8
Regional	302 (75%)	102 (25%)	2225	4.6	3.8–5.6
Metastatic	15 (25%)	44 (75%)	195	22.6	16.8–30.3
Smoking history/161					
Yes	26 (50%)	26 (50%)	356	7.3	5.0–10.7
No	864 (79%)	228 (21%)	6942	3.3	2.9–3.7
Alcohol-use history/162					
Yes	68 (72%)	27 (28%)	672	4.0	2.8–5.9
No	824 (79%)	224 (21%)	6636	3.4	3.0–3.8

*Per 100 PYFU = persons per year of follow-up; CI = confidence interval; BMI = body mass index; /# = missing values.

After stratification based on the cancer stages, the group diagnosed with metastatic disease had a mortality rate of 22.6 per 100 PYFU (95% CI: 16.8–30.3). Furthermore, the mortality patterns among individuals with a history of smoking and/or alcohol use revealed heightened mortality rates (7.3 per 100 PYFU; 95%CI: 5.0–10.7 and 4.0 per 100 PYFU; 95%CI: 2.8–5.9, respectively).

### Survival probabilities

**Fig 1:**
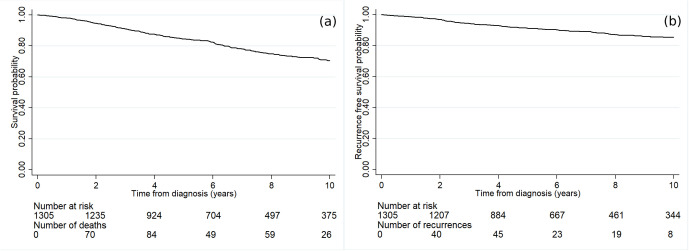
The outcome of luminal A breast cancer patients. **(a)** Survival rates **(b)** recurrence-free survival rates.

Fig 1a demonstrates the survival curve of luminal A breast cancer patients, portraying a gradual decrement in survival probability over time. The survival rates at 2, 4-, 6-, 8-, and 10-years post-diagnosis were 95%, 88%, 82%, 75%, and 70%, respectively.

**Fig 2:**
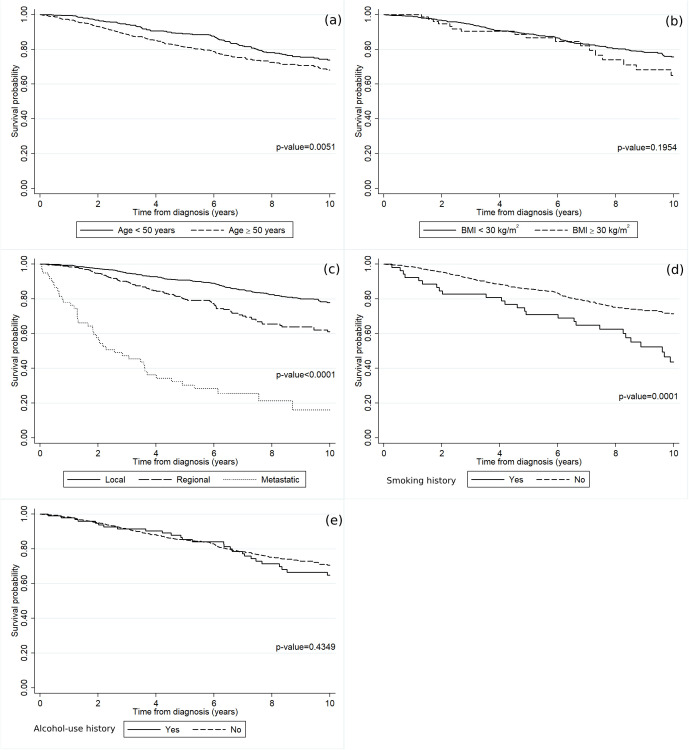
The survival rates of the luminal A breast cancer patients according to (a) age, (b) BMI, (c) cancer stage, (d) smoking history, and (e) alcohol-use history.

In Fig 2, the OS rates of luminal A breast cancer patients are stratified by fundamental baseline characteristics encompassing age, BMI, cancer stage, smoking history, and alcohol-use history. As can be seen in Fig 2a, patients aged ≥50 years old exhibited a markedly diminished survival rate in comparison to their younger counterparts. Furthermore, luminal A breast cancer patients diagnosed with the metastatic stage manifested a significantly lower survival rate relative to those diagnosed with either the regional or localized stage (Fig 2c). Notably, individuals with a history of smoking demonstrated a notably lower survival rate than their non-smoking counterparts (Fig 2d).

**Fig 3:**
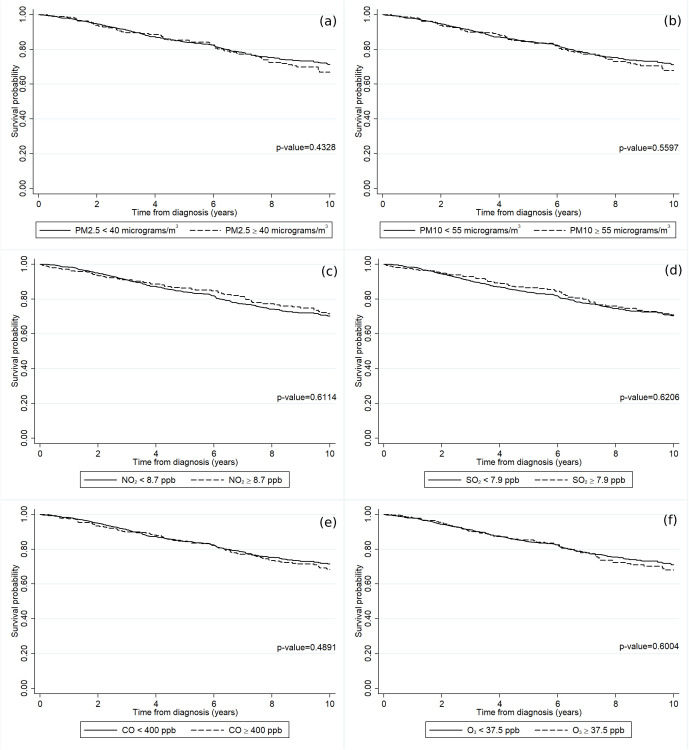
The survival rates of the luminal A breast cancer patients according to the annually averaged concentrations of (a) PM_2.5_, (b) PM_10_, (c) NO_2_, (d) SO_2_, (e) CO, and (f) O_3_.

Fig 3 shows the impact of air pollution on the survival rates predicated on the patients’ residences at the time of diagnosis. No differences in survival rates were observed among patients residing in areas according to the type of pollutant: PM_2.5_ (Fig 3a), PM_10_ (Fig 3b), NO_2_ (Fig 3c), SO_2_ (Fig 3d), CO (Fig 3e), and O_3_ (Fig 3f).

### Risk factors associated with death

Cox proportional hazard models were utilized to elucidate the pertinent risk factors contributing to mortality within the cohort of luminal A breast cancer patients, the results of which are available in Table 2. The last iteration of the multivariable analysis affirmed that age, cancer stage, and smoking history maintained significant associations with mortality risk (*p*-value<0.05). Of particular significance is that the metastatic cancer stage attained the highest adjusted hazard ratio (aHR) of 10.50 (95%CI: 7.23–15.25). Furthermore, the regional cancer stage (aHR = 2.04; 95%CI: 1.55–2.69), an age of ≥ 50 years old (aHR = 1.46; 95%CI: 1.13–1.90), and a history of smoking (aHR = 1.72; 95%CI: 1.14–2.60) all emerged as important factors augmenting the mortality risk within the luminal A breast cancer patient cohort.

**Table 2:** Risk factors associated with mortality among the luminal A breast cancer patients.

Variable			Univariable Analysis			Multivariable Analysis		
	Mortality	Total	HR	95% CI	*p*-value	aHR	95%CI	*p*-value
**At diagnosis**								
Age ≥ 50 years old	184	775	1.41	1.11–1.79	0.0048	1.46	1.13-1.90	0.0038
BMI ≥ 30 kg/m^2^	17	75	1.39	0.84–2.30	0.2170	–	–	–
Regional cancer stage	102	404	2.05	1.58–2.65	<0.0001	2.04	1.55–2.69	<0.0001
Metastatic cancer stage	44	59	10.75	7.61–15.18		10.50	7.23–15.25	
Smoking history	26	52	2.18	1.45–3.28	0.0007	1.72	1.14–2.60	<0.0001
Alcohol-use history	27	95	1.17	0.79–1.75	0.4449	–	–	–
**Time-updated variables**								
Residential concentration of PM_2.5 _≥ 40 (µg/m^3^)	–	–	1.12	0.84–1.50	0.4383	–	–	–
Residential concentration of PM_10_ ≥ 55 (µg/m^3^)	–	–	1.09	0.82–1.44	0.5629	–	–	–
Residential concentration of NO_2_ ≥ 8.7 ppb	–	–	0.93	0.70–1.23	0.6090	–	–	–
Residential concentration of SO_2_ ≥ 7.9 ppb	–	–	0.93	0.71–1.23	0.6185	–	–	–
Residential concentration of CO ≥ 400 ppb	–	–	1.09	0.85–1.41	0.4917	–	–	–
Residential concentration of O_3_ ≥ 37.5 ppb	–	–	1.07	0.82–1.40	0.6026	–	–	–

*p-value from a partial likelihood ratio test; HR = hazard ratio; CI = confidence interval; aHR = adjusted hazard ratio.

### Baseline characteristics and recurrence rate

Table 3 shows the recurrence patterns observed among 135 luminal A breast cancer patients. The cumulative recurrence rate for this cohort was computed as 1.7 per 100 PYFU (95%CI: 1.4–2.0). Furthermore, the recurrence rate for patients aged < 50 years old exhibited was 1.8 per 100 PYFU (95%CI: 1.4–2.3), while that for a high BMI was higher at 3.4 per 100 PYFU (95%CI: 2.0–5.6). Upon stratification by cancer stage, the metastatic stage group exhibited the highest recurrence rate of 7.7 per 100 PYFU (95%CI: 4.5–13.3). Furthermore, patients with a history of smoking and/or alcohol use demonstrated elevated recurrence rates of 3.2 per 100 PYFU (95%CI: 1.7–5.8) and 2.5 per 100 PYFU (95%CI: 1.6–4.1), respectively.

**Table 3:** Baseline characteristics and the associated cancer recurrence rate for the study population.

Characteristics	Survival (n (%))	Recurrence (n (%))	PYFU	Recurrence Rate	95%CI
Overall	1,170 (90%)	135 (10%)	8045	1.7	1.4–2.0
Age at diagnosis (years old)					
< 50	466 (88%)	64 (12%)	3523	1.8	1.4–2.3
≥ 50	704 (91%)	71 (9%)	4522	1.6	1.2–2.0
BMI (kg/m^2^)/415					
< 30	735 (90%)	79 (10%)	5185	1.5	1.2–1.9
≥ 30	60 (80%)	15 (20%)	445	3.4	2.0–5.6
Cancer stage/22					
Local	739 (90%)	81 (10%)	5612	1.4	1.2–1.8
Regional	364 (90%)	40 (10%)	2159	1.9	1.4–2.5
Metastatic	46 (78%)	13 (22%)	168	7.7	4.5–13.3
Smoking history/161					
Yes	42 (81%)	10 (19%)	320	3.2	1.7–5.8
No	975 (89%)	117 (11%)	6678	1.8	1.5–2.1
Alcohol-use history/162					
Yes	79 (83%)	16 (17%)	630	2.5	1.6–4.1
No	938 (90%)	110 (10%)	6378	1.7	1.4–2.1

*per 100 PYFU = persons per year of follow-up; CI = confidence interval; BMI = body mass index; /# = missing values.

### Recurrence-free survival probabilities

**Fig 4:**
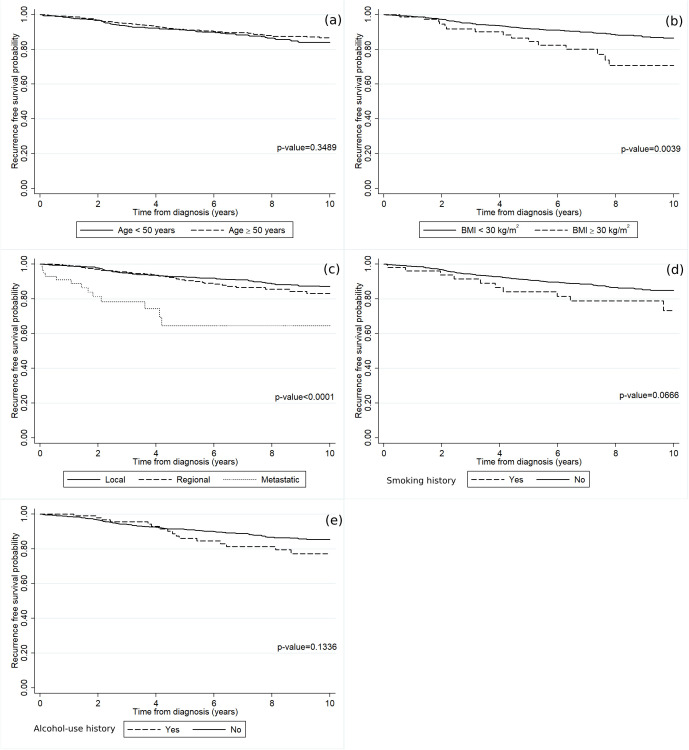
The recurrence-free survival rates of the luminal A breast cancer patients according to (a) age, (b) BMI, (c) cancer stage, (d) smoking history, and (e) alcohol-use history.

**Fig 5:**
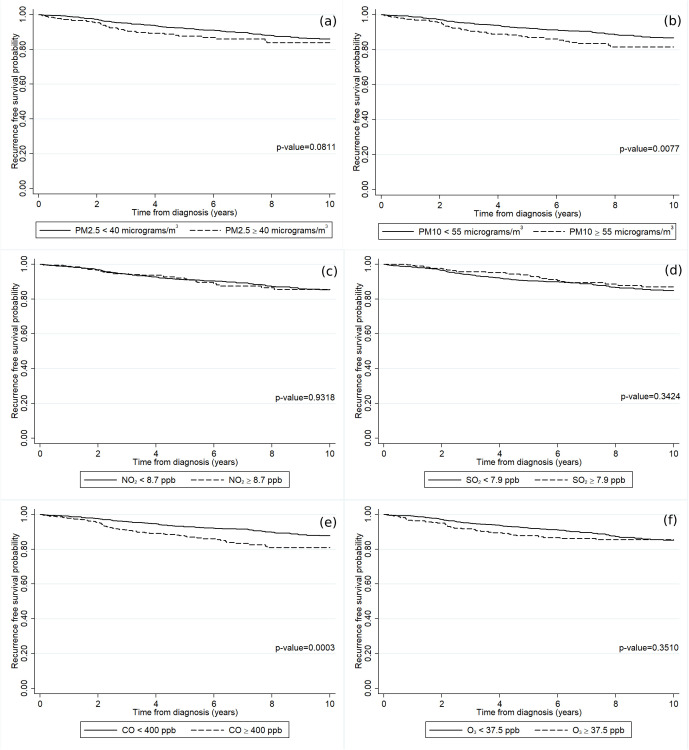
The recurrence-free survival rates of the luminal A breast cancer patients according to the annually averaged concentrations of (a) PM_2.5_, (b) PM_10_, (c) NO_2_, (d) SO_2_, (e) CO, and (f) O_3_.

The RFS rate among the luminal A breast cancer patients gradually declined over time as shown in Fig 1b. The RFS rate at 2, 4, 6, 8, and 10 years were 97%, 93%, 90%, 87%, and 85%, respectively. Fig 4 demonstrates the RFS rate of luminal A breast cancer patients across the various baseline characteristics included in the study. Fig 4b unveils a notable reduction in the RFS rate among patients with a high BMI. In addition, the luminal A breast cancer patients diagnosed with the metastatic stage exhibited a significantly diminished RFS rate in comparison to those diagnosed with either the regional or localized stage (Fig 4c). Fig 5 shows the temporal effects of air pollution on the cancer RFS rate based on the patients’ residences. Statistically significant effects were found for patients who had high annual averaged concentrations of PM_10_ (Fig 5b) and CO (Fig 5e).

### Risk factors associated with luminal A breast cancer recurrence

The findings delineating risk factors pertinent to recurrence risk among luminal A breast cancer patients derived from Cox proportional hazard models are provided in Table 4. In the multivariable analysis, pollutants correlated with PM_10_ concentration were deliberately excluded, thereby refining the precision of the model. Subsequently, only cancer stage, smoking history, and the time-updated local concentration of PM_10_ were included in the multivariate framework. In the ultimate model, all the parameters except for smoking history demonstrated independent associations with mortality risk (*p*-values < 0.05). Metastatic cancer stage attained the highest aHR of 4.96 (95%CI: 2.78–8.83). Furthermore, exposure to the time-updated local concentration of PM_10_ above 55 µg/m^3^ was found to be linked with an augmented recurrence risk among the luminal A breast cancer patients (aHR = 1.68; 95%CI: 1.16–2.45). Maps that represent trends in PM_10_ levels over time shown in S2 Fig.

**Table 4:** Risk factors associated with recurrence among the luminal A breast cancer patients.

Variable			Univariable Analysis			Multivariable Analysis		
	Recurrence	Total	HR	95%CI	*p*-value	aHR	95%CI	*p*-value
**At diagnosis**								
Age ≥ 50 years old	71	775	0.85	0.61–1.19	0.3504	–	–	–
BMI ≥ 30 kg/m^2^	15	75	2.21	1.27–3.84	0.0102	–	–	–
Metastatic cancer stage	13	59	4.96	2.78–8.84	<0.0001	4.96	2.78–8.83	<0.0001
Smoking history	10	52	1.81	0.95–3.46	0.0958	1.55	0.81–2.97	0.1840
Alcohol-use history	16	95	1.49	0.88–2.52	0.1562	–	–	–
**Time-updated variables**								
Residential concentration of PM_2.5 _≥ 40 (µg/m^3^)	–	–	1.42	0.96–2.12	0.0927	–	–	–
Residential concentration of PM_10_ ≥ 55 (µg/m^3^)	–	–	1.65	1.14–2.40	0.0111	1.68	1.16–2.45	0.0060
Residential concentration of NO_2_ ≥ 8.7 ppb	–	–	1.02	0.68–1.52	0.9319	–	–	–
Residential concentration of SO_2_ ≥ 7.9 ppb	–	–	0.82	0.54–1.24	0.3339	–	–	–
Residential concentration of CO ≥ 400 ppb	–	–	1.88	1.33–2.65	0.0004	–	–	–
Residential concentration of O_3_ ≥ 37.5 ppb	–	–	1.20	0.82–1.76	0.3586	–	–	–

*p-value from a partial likelihood ratio test. HR = hazard ratio; CI = confidence interval; aHR = adjusted hazard ratio.

### Modeling lagged (delayed) associations between air pollution exposure and the mortality and recurrence of luminal A breast cancer

**Fig 6:**
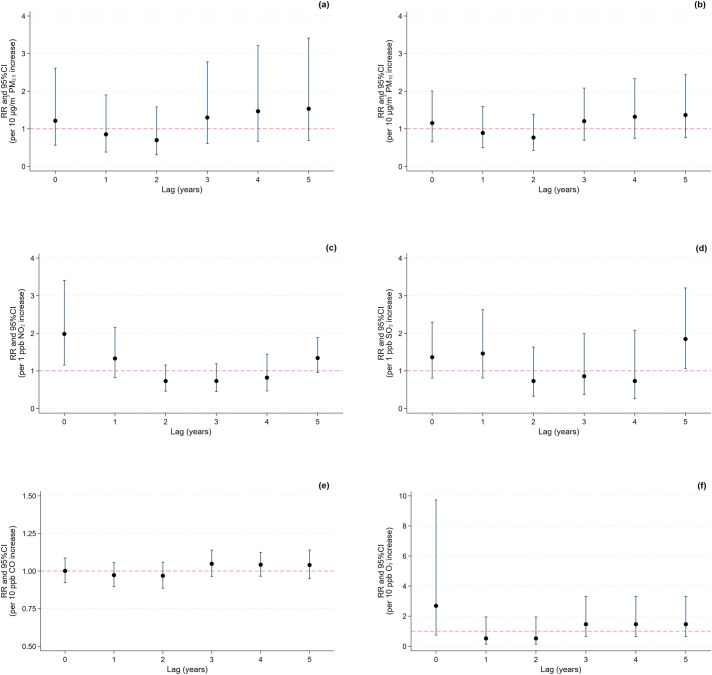
Lag-specific relative risks of luminal A breast cancer-associated mortality per 10-unit increase in annual concentrations of (a) PM_2.5_, (b) PM_10_, (e) CO, and (f) O_3_ and per 1-unit increase in annual concentrations of (c) NO_2_ and (d) SO_2_.

**Fig 7:**
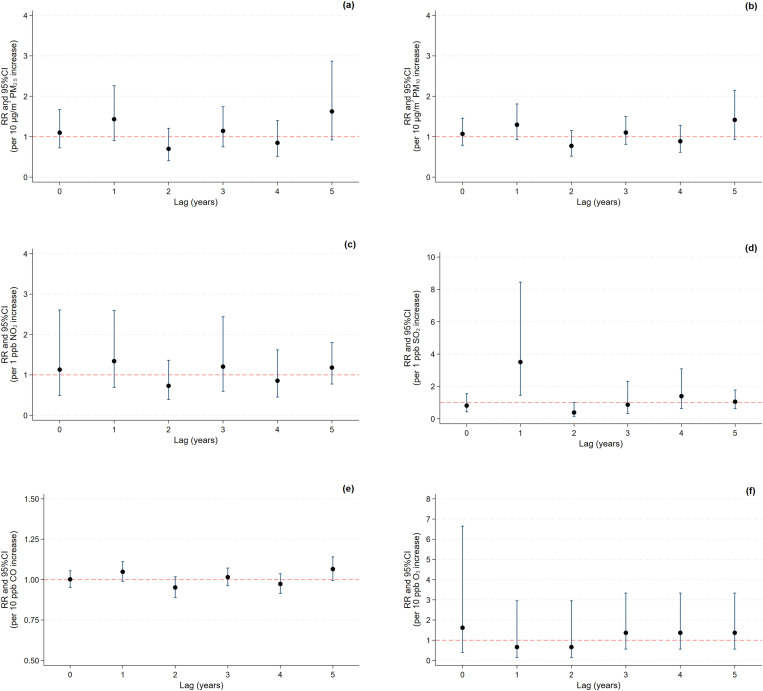
Lag-specific relative risks of luminal A breast cancer recurrence per 10-unit increase in annual concentrations of (a) PM_2.5_, (b) PM_10_, (e) CO, and (f) O3 and per 1-unit increase in annual concentrations of (c) NO_2_ and (d) SO_2_.

Lag 5 shows the lowest values for AIC (11.00), HQ (10.80), and SBIC (11.95), and the sequential modified LR test suggests that a 5-lag specification is optimal (S1 Table). Therefore, we selected lag 5 as the optimal lag length, indicating superior overall model performance. RRs with 95% CIs for recurrence and mortality outcomes for luminal A breast cancer per unit increase in air pollutant concentration were estimated using a distributed lag model (S2 Table). An increase in NO_2_ concentration of 1 ppb was associated with a higher risk of total deaths at lag 0 years (RR: 1.98, 95% CI: 1.15–3.41) (Fig 6c). A 1 ppb increase in SO_2_ concentration at lag 1 year was significantly associated with a 3.5-fold increase in recurrence risk (RR: 3.51, 95% CI: 1.45–8.45) (Fig 7d), while a 1 ppb increase in SO_2_ concentration at lag 5 years was associated with a higher mortality risk (RR: 1.85, 95% CI: 1.06–3.21) (Fig 6d). However, the cumulative risk (lag 0–5 years) per 10-unit increase in annual concentrations of PM_2.5_, PM_10_, CO, and O_3_, and per 1-unit increase in annual concentrations of NO_2_ and SO_2_ was not associated with mortality from and recurrent luminal A breast cancer (S3 Fig).

## Discussion

Existing studies have primarily focused on widely-known risk factors of luminal A breast cancer such as age, alcohol consumption, smoking habit, BMI, and stage of cancer [11–16], but not much about the effects of air pollution has been explored. Since luminal A breast cancer has favorable prognosis with many long-term survivors [6,7], analyzing the impact of prolonged exposure to air pollutants on the recurrence and survival risks of these patients is necessary for the improvements of healthcare and public health strategies. In the present study, we explored potential correlations between factors that could affect luminal A breast cancer 10-year recurrence rates and mortality of a cohort of patients in the upper northern region of Thailand. While we established a connection between exposure to air pollution and luminal A breast cancer mortality/recurrence risks, it is important to emphasize that we identified an association rather than a causal relationship. The findings suggest that patients living in areas with higher levels of PM_10_ exposure had shorter recurrence-free times. However, we cannot definitively conclude that air pollution directly caused these outcomes. This distinction is critical to avoid insinuating any causality and provide a more balanced interpretation of our findings. The study encompassed all women who had been diagnosed with luminal A breast cancer in Thailand during 2003–2018. Most of the luminal A breast cancers were of the local stage (62.8%; 820/1,305 cases), which is in line with it frequently having a favorable prognosis relative to other subtypes attributable to its characteristic traits of restrained growth rate and consistent responsiveness to hormone therapy.

From a study conducted in China with a median follow-up of 5.7 years, luminal A tumors had RFS and OS rates of 87.3% and 94.2%, respectively [43]. In comparison, we determined 10-year RFS and OS rates of 85% and 70%, respectively. Luminal A breast cancer could have a greater tendency to recur later so at least 10 years of follow-up when studying recurrence is important [7,25,44,45]. Ten-year RFS of Dutch study was 87.5% which was in line with our study [7].

We discovered that patients diagnosed with luminal A breast cancer aged 50 years old or older face a mortality rate risk approximately 1.5 times higher than those below this age. These observations align with those from earlier research [10], implying an association between luminal A breast cancer and an augmented risk of death in older women. Moreover, we found that patients with the regional or metastatic cancer stage exhibited a significantly elevated risk of death (aHR: 2.04; 95%CI: 1.55–2.69 vs. aHR:10.50; 95%CI: 7.23–15.25). Moreover, our findings also reveal that patients with both luminal A breast cancer and a history of smoking had a mortality rate risk of approximately 1.7 times greater. This observation agrees with recent investigations conducted on women aged 20–69 years old diagnosed with primary invasive luminal breast cancer in the Seattle–Puget Sound region [46], in which the risk of mortality rate was 1.6 times higher. Our study is no significant difference between the mortality rate among patients residing in high pollution. This observation is inconsistent with Cheng et al. [20] found that PM_2.5_ and PM_10_ (per 10 µg/m^3^) had a mortality rate risk of approximately 1.17 and 1.13 times, respectively, including the study of Hwang et al. [19] that found PM_10_ (per 10 µg/m^3^) associated with breast cancer mortality rate about 1.05 times. However, we could not perform our analysis at WHO guidance levels since the PM data are left-skewed. In other words, the PM in our study areas were higher than the standard levels. Therefore, in our analysis, we grouped them using quartiles and by choosing a suitable dichotomization.

In the context of recurrence rates, we found that 85% of luminal A breast cancer patients survived 10 years post-diagnosis regardless of the residential concentration of air pollution. This finding is consistent with that of Mudduwa et al. [47], who discovered that 83.6% of luminal A breast cancer cases in Southern Sri Lanka from 2006 to 2015 were recurrence-free over a five-year post-diagnosis period. However, our results uncover a noteworthy disparity in recurrence times among luminal A breast cancer patients residing in districts with annually averaged PM_10_ concentrations either below or equal to or above 55 µg/m^3^. Specifically, a lower recurrence-free rate was observed when PM_10_ levels were equal to or exceeded 55 µg/m^3^. Thus, individuals residing in areas severely impacted by high levels of air pollution experienced a shorter recurrence-free time. To the best of our knowledge, the relationship between air pollution and recurrence rates in luminal A breast cancer patients has not previously been explored. It is imperative to emphasize that luminal A breast cancer patients constitute a vulnerable group residing in areas with PM_10_ concentrations surpassing the WHO guidance level. Our findings underscore the critical necessity to address the severe issue of air pollution, particularly in northern Thailand. To our knowledge, this is first study to evaluate the effects of air pollution and the RFS rate among the luminal A breast cancer patients, so it is a strength of this study.

Our findings on air pollution exposure and risk of mortality and recurrence indicate that a 1 ppb increase in NO_2_ was associated with a 1.98-fold increase in mortality within the same year. This suggests that in the short term, higher NO_2_ levels are associated with an elevated mortality risk. This is in keeping with Reding et al. [22], who found that NO_2_ increased the risk of ER + /PR+ breast cancer, with an RR of 1.10 (95% CI: 1.02–1.19 per IQR of 5.8 ppb) using a Land use regression model. Furthermore, we discovered that SO_2_ was associated with an increase in recurrence risk at lag 1 year and mortality risk at lag 5 years. This finding is consistent with Hwang et al. [19], who found that an increment in SO_2_ concentration of 1ppb increased the risk of breast cancer (OR = 1.04; 95% CI: 1.02–1.05). Conversely, Khorrami et al. [48] found that the concentration of SO_2_ inversely affected the severity of breast cancer among women over 50 years old. However, these study populations were not limited to luminal A breast cancer patients. Understanding the delayed effects of air pollution exposure enhances the ability to assess long-term health risks and provides crucial information for future surveillance and healthcare management of luminal A breast cancer patients.

The study’s findings have important implications for policies concerning pollution and patient care. The association between higher air pollution levels and the recurrence of luminal A breast cancer underscores the importance of addressing air quality in regions with high pollution, particularly in northern Thailand. Policymakers should consider the potential health impacts of air pollution on vulnerable populations such as breast cancer patients, and they might want to explore air quality regulations or stricter pollution controls to mitigate these risks. For healthcare providers, these results suggest that breast cancer patients living in high-pollution areas might benefit from additional lifestyle recommendations, such as reducing outdoor activities during high-pollution periods, utilizing air filtration systems at home, or considering other preventive measures that could reduce exposure to environmental pollutants. For clinical practice, we recommend adapting breast cancer follow-up protocols to include environmental risk stratification. Breast cancer patients residing in high-pollution areas should undergo more frequent clinical evaluations. This should be accompanied by comprehensive health education for patients, caregivers, and healthcare personnel on the signs and symptoms of breast cancer recurrence, including both locoregional and distant recurrence, to facilitate earlier detection. Additionally, primary care providers should be trained to consider environmental exposure history when planning long-term management and survivorship care. To effectively apply these findings to regions with different air pollution profiles, it would be beneficial to conduct comparative studies across different regions and analyze area-specific pollutant compositions. Furthermore, consideration should be given to local environmental and demographic factors, along with the potential for meta-analyses that combine data from multiple regions. This comprehensive approach would provide a more robust understanding of how air pollution impacts health outcomes across different geographical and environmental contexts.

Our study has some limitations. First, this study was conducted hospital-based nature, which may potentially compromise the generalizability of the results. However, this concern was addressed by the 10-year follow-up period with a commendable completeness rate of 73.6% (961/1,305). Moreover, the tumor grade, histology, and treatment for each luminal A breast cancer patient, while present in hospital medical records, were not systematically linked with the Chiang Mai Cancer Registry. Consequently, we were unable to investigate these factors in our analysis. If they had been linked, we could have conducted a more comprehensive analysis that included exploring the influence of tumor grade, histology, and treatment on luminal A breast cancer patient recurrence and mortality. Finally, due to a 10-year patient follow-up period, we were unable to examine associations with exposure lags exceeding 5 years as this would have rendered the lag analysis unreliable. In addition, the low number of deaths and recurrences observed annually in luminal-A breast cancer cases resulted in wide 95% CI when applying Poisson regression models and distributed lag models. Therefore, future studies should consider employing these methodologies to larger sample sizes to enhance the precision of the analysis.

## Conclusion

Not only BMI and cancer stage, but exposure to PM_10_ and CO had also adverse associations to recurrence risk in luminal A breast cancer patients in upper northern Thailand. An elevated breast cancer recurrence risk was associated with the time-updated local concentration of PM_10_ over 55 µg/m^3^. These findings are crucial for encouraging public health and allows the people who are related in the decision-making process to consider the women health impacts. While we identified an association between air pollution and luminal A breast cancer recurrence/mortality, further research is needed to establish whether a causal relationship exists. Emphasizing that there is an association rather than a causal relationship ensures a more balanced and cautious interpretation of our findings, thereby guiding future studies in this area.

## Supporting information

S1 Fig Annual average concentrations of (a) PM_2.5_, (b) PM_10_, (c) NO_2_, (d) SO_2_, (e) CO, and (f) O_3_. (DOCX)

S2 Fig PM_10_ levels in the study area from 2003–2020. This map was created with QGIS using open data (shapefile) from the Geo-Informatics and Space Technology Development Agency (https://gistdaportal.gistda.or.th/data/rest/services/EEC_Public).
(DOCX)

S3 Fig Cumulative relative risks (lag 0–5 years) for mortality from (a) and recurrence of (b) luminal A breast cancer per 10-unit increase in annual concentrations of PM_2.5_, PM_10_, CO, and O_3_ and per 1-unit increase in annual concentrations of NO_2_ and SO_2_. (DOCX)

S1 Table Lag order selection criteria. (DOCX)

S2 Table Relative risks (RRs) with 95% confidence intervals (CIs) for luminal A breast cancer-associated deaths and recurrences with per unit increase in air pollutant concentrations estimated using a distributed lag model. (DOCX)
