# Supplementary figures and images for: The recurrence and mortality risk in Luminal A breast cancer patients who lived in high pollution area
Source: PLoS One. 2025 Oct 17;20(10):e0335140. doi: 10.1371/journal.pone.0335140 (PMC12533841; doi:10.1371/journal.pone.0335140)

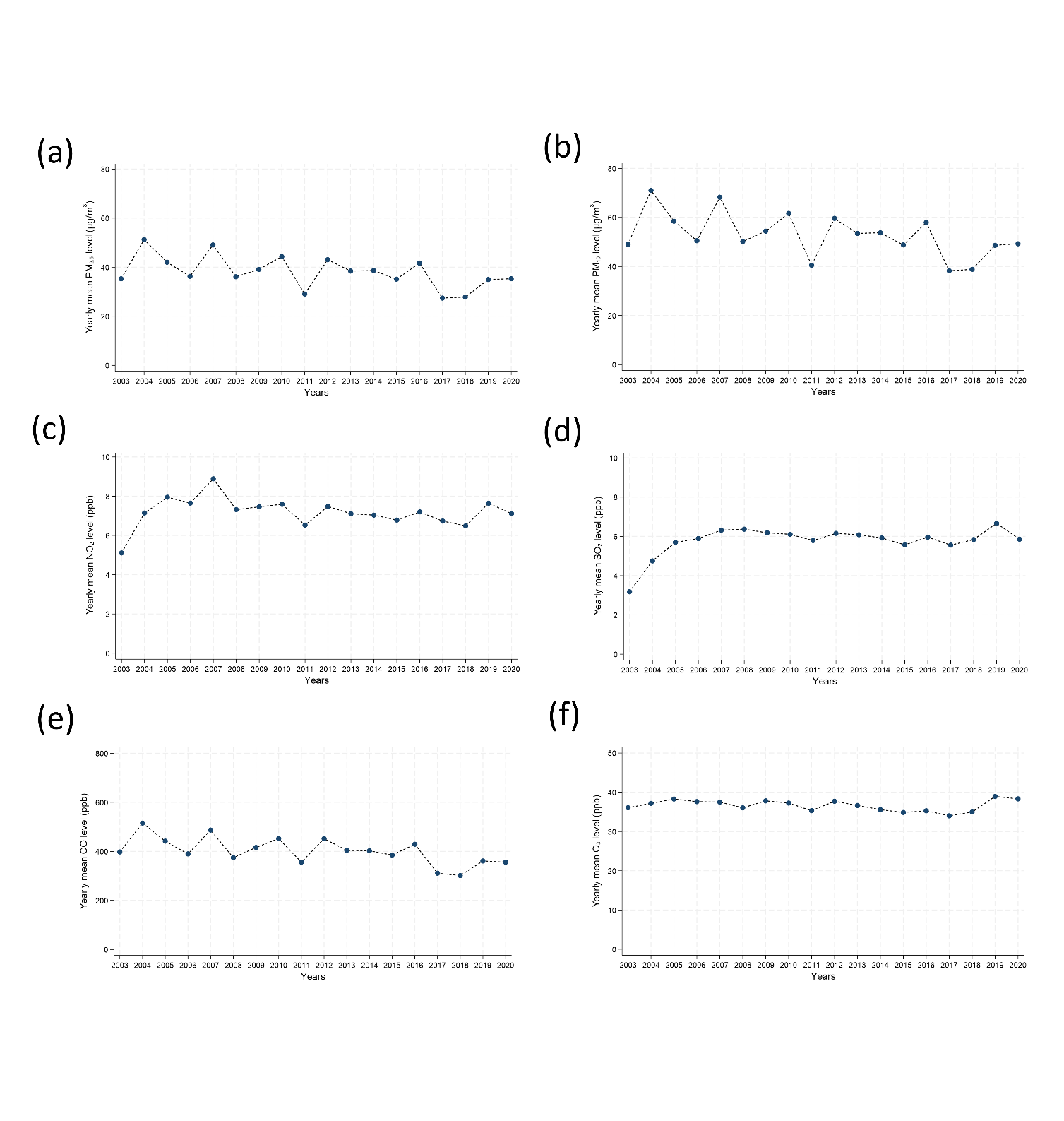


**S1 Fig.** **Annual average concentrations of (a) PM2.5, (b) PM10, (c) NO2, (d) SO2, (e) CO, and (f) O3.**

Supplement: S1 Fig — (DOCX) [file pone.0335140.s001.docx]
