# Supplementary material for: The recurrence and mortality risk in Luminal A breast cancer patients who lived in high pollution area
Source: PLoS One. 2025 Oct 17;20(10):e0335140. doi: 10.1371/journal.pone.0335140 (PMC12533841; doi:10.1371/journal.pone.0335140)
Supplement: S2 Fig — This map was created with QGIS using open data (shapefile) from the Geo-Informatics and Space Technology Development Agency (https://gistdaportal.gistda.or.th/data/rest/services/EEC_Public). (DOCX) [file pone.0335140.s002.docx]

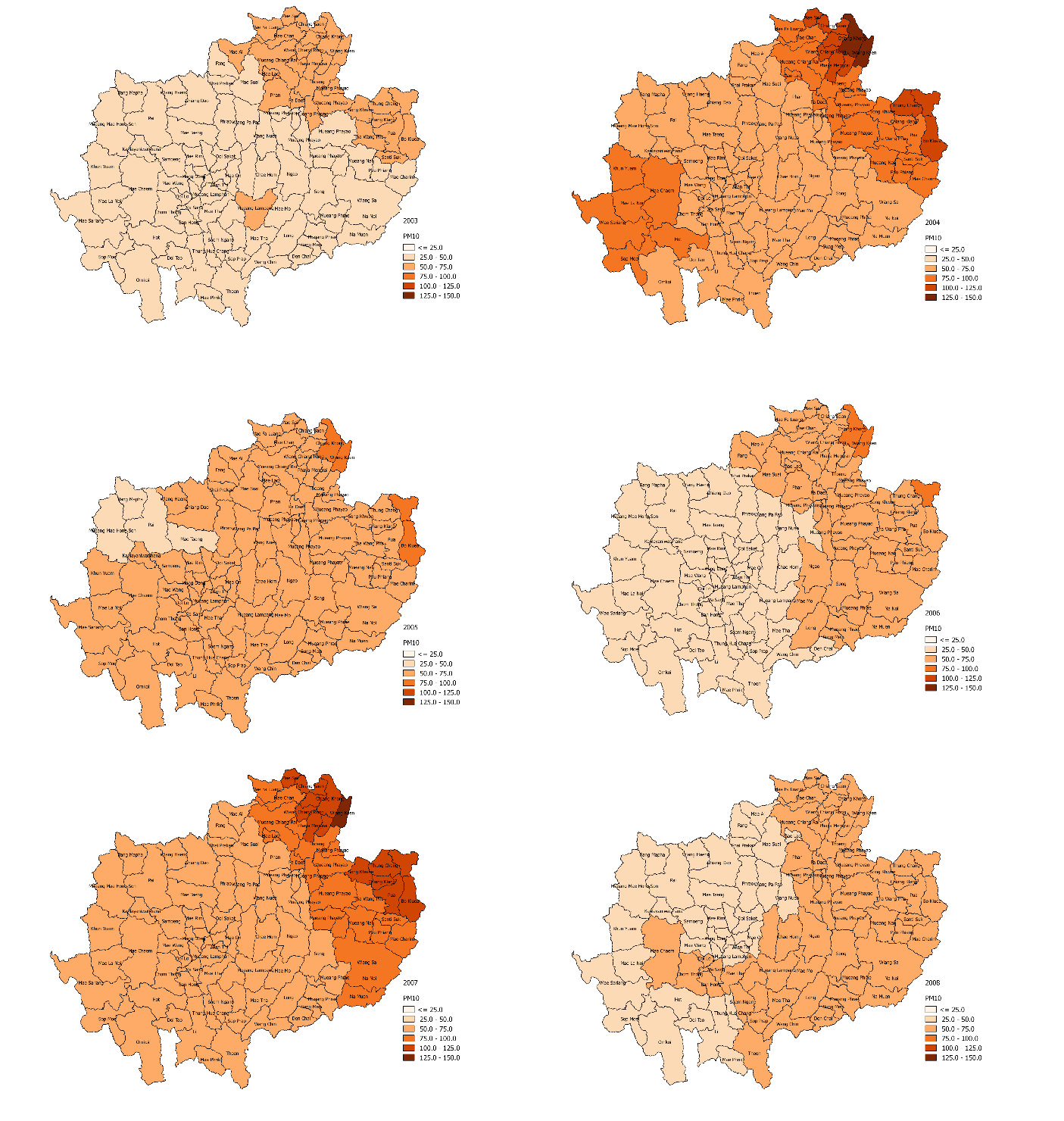


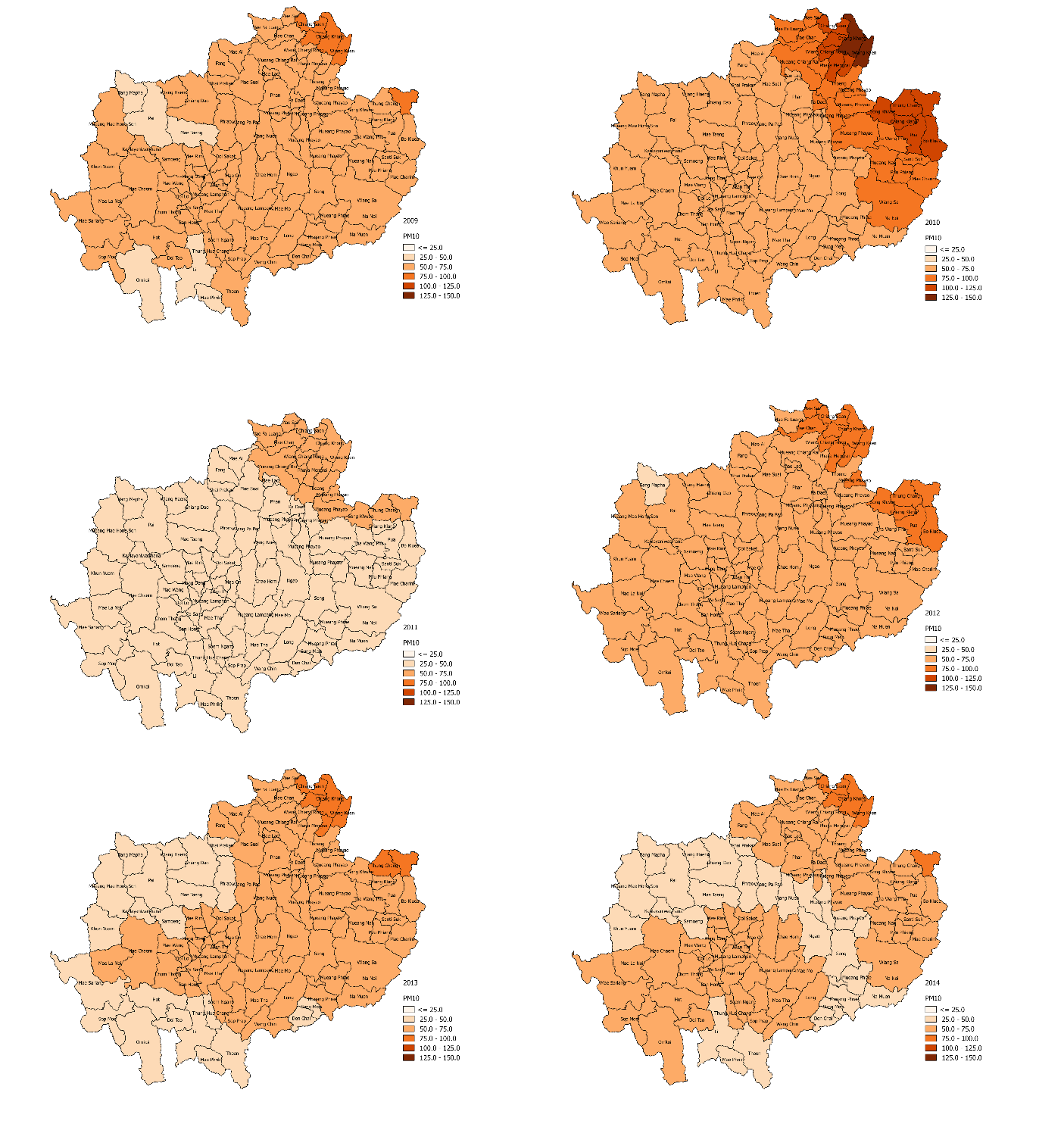


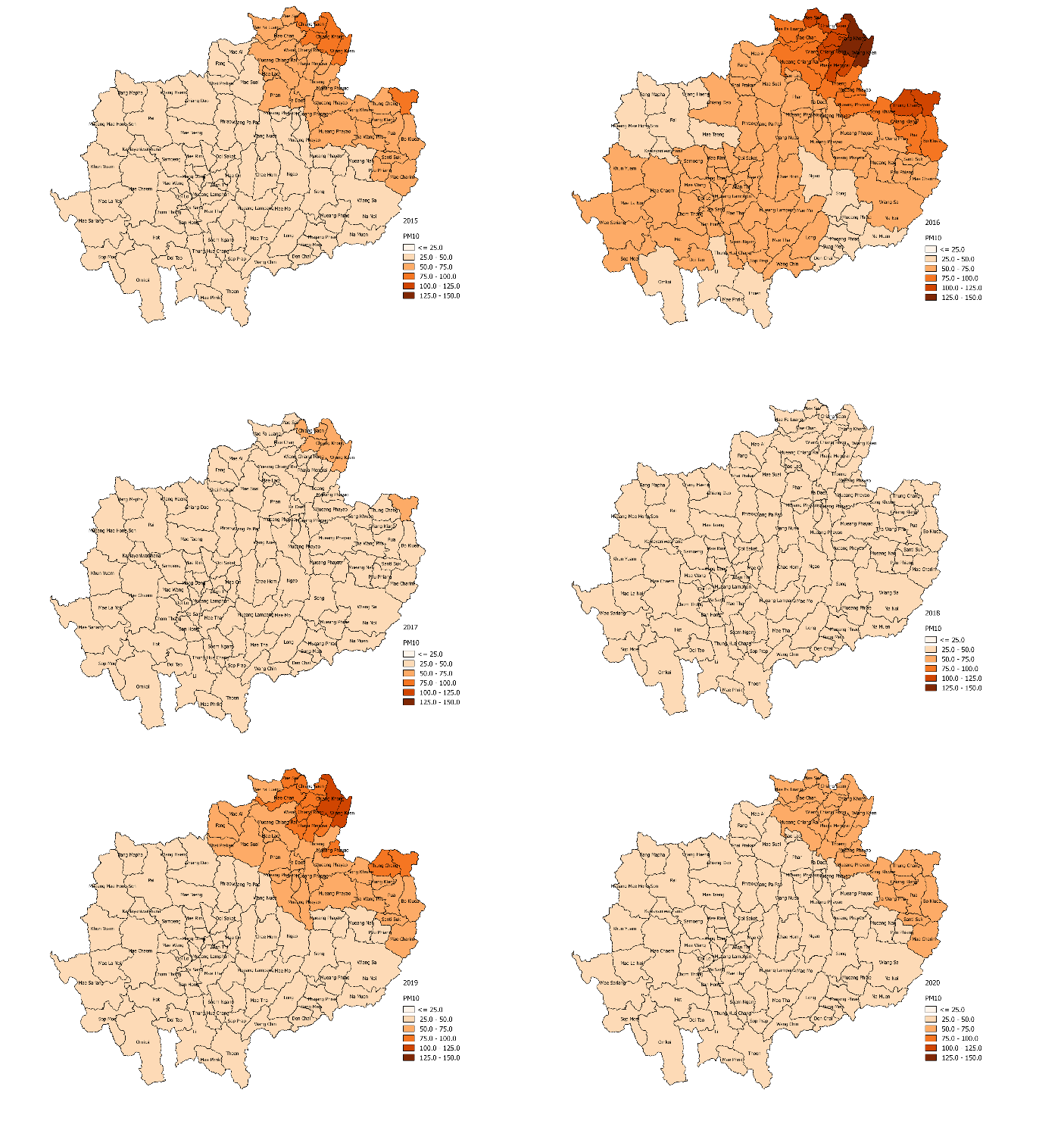


**S2 Fig.** **PM_10_ levels in the study area from 2003–2020. This map was created with QGIS using open data (shapefile) from the Geo-Informatics and Space Technology Development Agency (**[**https://gistdaportal.gistda.or.th/data/rest/services/EEC_Public).**](https://gistdaportal.gistda.or.th/data/rest/services/EEC_Public).)
