# Supplementary material for: The recurrence and mortality risk in Luminal A breast cancer patients who lived in high pollution area
Source: PLoS One. 2025 Oct 17;20(10):e0335140. doi: 10.1371/journal.pone.0335140 (PMC12533841; doi:10.1371/journal.pone.0335140)
Supplement: S3 Fig — (DOCX) [file pone.0335140.s003.docx]

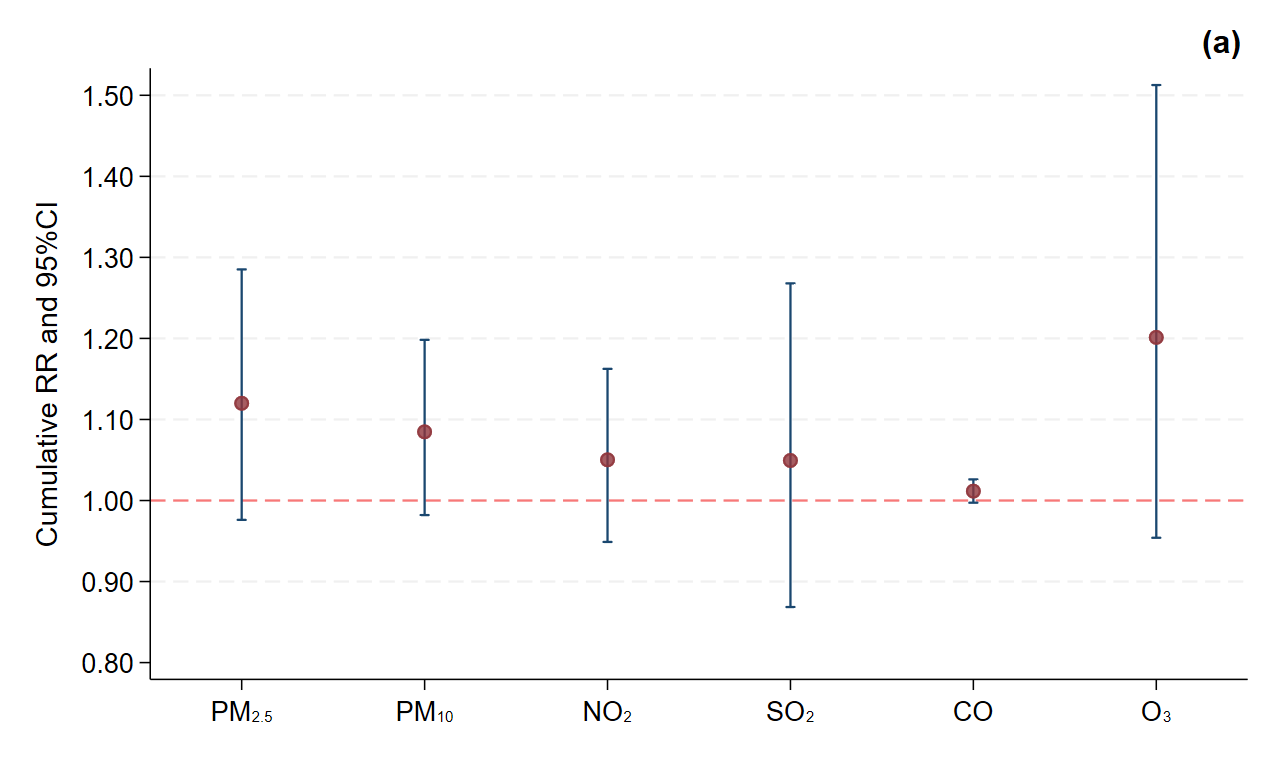


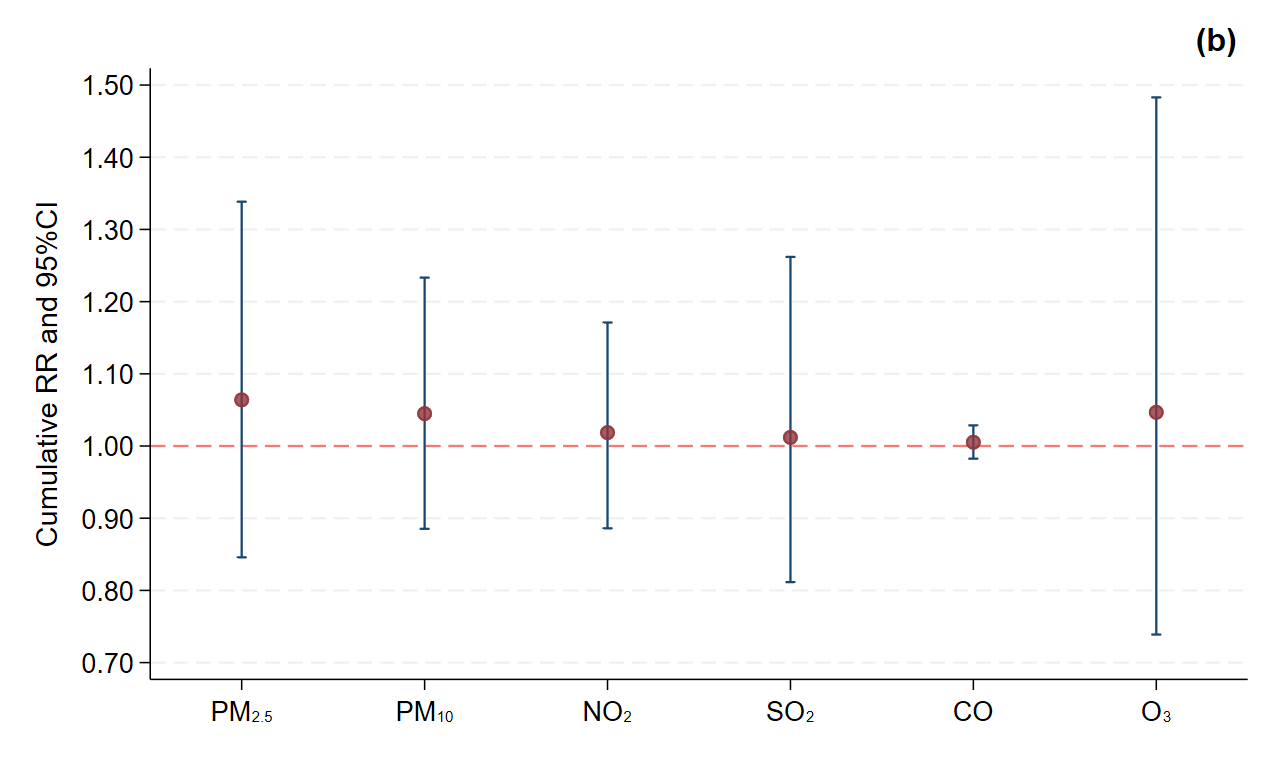


**S3 Fig** Cumulative relative risks (lag 0–5 years) for mortality from (a) and recurrence of (b) luminal A breast cancer per 10-unit increase in annual concentrations of PM_2.5_, PM_10_, CO, and O_3_ and per 1-unit increase in annual concentrations of NO_2_ and SO_2_.
