# Supplementary material for: The recurrence and mortality risk in Luminal A breast cancer patients who lived in high pollution area
Source: PLoS One. 2025 Oct 17;20(10):e0335140. doi: 10.1371/journal.pone.0335140 (PMC12533841; doi:10.1371/journal.pone.0335140)
Supplement: S1 Table — (DOCX) [file pone.0335140.s004.docx]

**S1 Table.**  **Lag order selection criteria**

| **Lag** | **LR (p-value)** | **FPE** | **AIC** | **HQ** | **SBIC** |
| --- | --- | --- | --- | --- | --- |
| 0 | NA | 1097.04 | 12.68 | 12.66 | 12.76 |
| 1 | 17.72 (0.001) | 527.91 | 11.93 | 11.87 | 12.19 |
| 2 | 9.43 (0.051) | 505.39* | 11.82 | 11.73 | 12.25 |
| 3 | 3.95 (0.413) | 818.72 | 12.13 | 12.00 | 12.74 |
| 4 | 12.87 (0.012) | 828.07 | 11.75 | 11.59 | 12.54 |
| 5 | 17.85 (0.001) | 998.83 | 11.00* | 10.80* | 11.95* |

LR: Sequential modified LR test statistic (each test at 5% level), FPE: Final prediction error, AIC: Akaike’s information criterion, HQ: Hannan-Quinn information criterion, SBIC: Schwarz's Bayesian information criterion, *Indicates lag order selected by the criteria.
