# Supplementary material for: The recurrence and mortality risk in Luminal A breast cancer patients who lived in high pollution area
Source: PLoS One. 2025 Oct 17;20(10):e0335140. doi: 10.1371/journal.pone.0335140 (PMC12533841; doi:10.1371/journal.pone.0335140)
Supplement: S2 Table — (DOCX) [file pone.0335140.s005.docx]

**S2 Table. Relative risks (RRs) with 95% confidence intervals (CIs) for luminal A breast cancer-associated deaths and recurrences with per unit increase in air pollutant concentrations estimated using a distributed lag model.**

| **Recurrence** | | | | | **Deaths** | | | | |
| --- | --- | --- | --- | --- | --- | --- | --- | --- | --- |
| **All Lag Terms Modeled Together** | | | | | **All Lag Terms Modeled Together** | | | | |
| **Lag (years)** | **RR** | **95% CI** | | ***p*-value** | **Lag (years)** | **RR** | **95% CI** | | ***p*-value** |
| **PM_2.5_** |  |  |  |  | **PM_2.5_** |  |  |  |  |
| 0 | 1.10 | 0.72 | 1.67 | 0.66 | 0 | 1.21 | 0.56 | 2.61 | 0.62 |
| 1 | 1.43 | 0.91 | 2.26 | 0.12 | 1 | 0.85 | 0.38 | 1.90 | 0.69 |
| 2 | 0.70 | 0.41 | 1.21 | 0.20 | 2 | 0.70 | 0.31 | 1.58 | 0.39 |
| 3 | 1.14 | 0.75 | 1.74 | 0.53 | 3 | 1.30 | 0.61 | 2.78 | 0.50 |
| 4 | 0.85 | 0.51 | 1.40 | 0.52 | 4 | 1.47 | 0.67 | 3.21 | 0.34 |
| 5 | 1.63 | 0.92 | 2.87 | 0.09 | 5 | 1.53 | 0.69 | 3.41 | 0.30 |
| Net effect | 1.06 | 0.85 | 1.34 | 0.60 | Net effect | 1.12 | 0.98 | 1.29 | 0.11 |
| **PM_10_** |  |  |  |  | **PM_10_** |  |  |  |  |
| 0 | 1.07 | 0.79 | 1.46 | 0.66 | 0 | 1.15 | 0.66 | 2.01 | 0.62 |
| 1 | 1.30 | 0.93 | 1.81 | 0.13 | 1 | 0.89 | 0.50 | 1.59 | 0.69 |
| 2 | 0.77 | 0.52 | 1.15 | 0.21 | 2 | 0.77 | 0.42 | 1.39 | 0.38 |
| 3 | 1.10 | 0.81 | 1.50 | 0.53 | 3 | 1.20 | 0.70 | 2.08 | 0.51 |
| 4 | 0.89 | 0.61 | 1.28 | 0.52 | 4 | 1.32 | 0.75 | 2.34 | 0.34 |
| 5 | 1.42 | 0.93 | 2.15 | 0.10 | 5 | 1.37 | 0.76 | 2.44 | 0.29 |
| Net effect | 1.04 | 0.89 | 1.23 | 0.60 | Net effect | 1.08 | 0.98 | 1.20 | 0.11 |
| **NO_2_** |  |  |  |  | **NO_2_** |  |  |  |  |
| 0 | 1.13 | 0.49 | 2.61 | 0.77 | 0 | 1.98 | 1.15 | 3.41 | 0.01 |
| 1 | 1.34 | 0.69 | 2.60 | 0.39 | 1 | 1.33 | 0.82 | 2.15 | 0.25 |
| 2 | 0.73 | 0.39 | 1.36 | 0.32 | 2 | 0.73 | 0.46 | 1.16 | 0.18 |
| 3 | 1.20 | 0.59 | 2.44 | 0.61 | 3 | 0.73 | 0.45 | 1.19 | 0.21 |
| 4 | 0.86 | 0.45 | 1.62 | 0.63 | 4 | 0.82 | 0.47 | 1.44 | 0.49 |
| 5 | 1.18 | 0.77 | 1.80 | 0.44 | 5 | 1.34 | 0.96 | 1.88 | 0.09 |
| Net effect | 1.02 | 0.89 | 1.17 | 0.80 | Net effect | 1.05 | 0.95 | 1.16 | 0.34 |
| **SO_2_** |  |  |  |  | **SO_2_** |  |  |  |  |
| 0 | 0.81 | 0.43 | 1.55 | 0.53 | 0 | 1.36 | 0.81 | 2.30 | 0.25 |
| 1 | 3.51 | 1.45 | 8.45 | 0.01 | 1 | 1.46 | 0.81 | 2.63 | 0.21 |
| 2 | 0.39 | 0.15 | 1.01 | 0.054 | 2 | 0.73 | 0.33 | 1.63 | 0.44 |
| 3 | 0.87 | 0.33 | 2.32 | 0.78 | 3 | 0.86 | 0.37 | 1.99 | 0.72 |
| 4 | 1.40 | 0.63 | 3.09 | 0.41 | 4 | 0.73 | 0.26 | 2.08 | 0.56 |
| 5 | 1.05 | 0.62 | 1.78 | 0.84 | 5 | 1.85 | 1.06 | 3.21 | 0.03 |
| Net effect | 1.01 | 0.81 | 1.26 | 0.92 | Net effect | 1.05 | 0.87 | 1.27 | 0.62 |
| **CO** |  |  |  |  | **CO** |  |  |  |  |
| 0 | 1.00 | 0.95 | 1.05 | 0.94 | 0 | 1.00 | 0.92 | 1.09 | 0.98 |
| 1 | 1.05 | 0.99 | 1.11 | 0.11 | 1 | 0.97 | 0.90 | 1.06 | 0.51 |
| 2 | 0.95 | 0.89 | 1.02 | 0.15 | 2 | 0.97 | 0.89 | 1.06 | 0.48 |
| 3 | 1.02 | 0.96 | 1.07 | 0.58 | 3 | 1.05 | 0.96 | 1.14 | 0.27 |
| 4 | 0.97 | 0.91 | 1.04 | 0.40 | 4 | 1.04 | 0.97 | 1.12 | 0.29 |
| 5 | 1.07 | 0.99 | 1.14 | 0.07 | 5 | 1.04 | 0.95 | 1.14 | 0.40 |
| Net effect | 1.01 | 0.98 | 1.03 | 0.65 | Net effect | 1.01 | 1.00 | 1.03 | 0.11 |
| **O_3_** |  |  |  |  | **O_3_** |  |  |  |  |
| 0 | 1.62 | 0.39 | 6.65 | 0.50 | 0 | 2.69 | 0.74 | 9.73 | 0.13 |
| 1 | 0.66 | 0.15 | 2.95 | 0.59 | 1 | 0.52 | 0.14 | 1.95 | 0.33 |
| 2 | 0.66 | 0.15 | 2.95 | 0.59 | 2 | 0.52 | 0.14 | 1.95 | 0.33 |
| 3 | 1.37 | 0.56 | 3.34 | 0.49 | 3 | 1.46 | 0.65 | 3.31 | 0.36 |
| 4 | 1.37 | 0.56 | 3.34 | 0.49 | 4 | 1.46 | 0.65 | 3.31 | 0.36 |
| 5 | 1.37 | 0.56 | 3.34 | 0.49 | 5 | 1.46 | 0.65 | 3.31 | 0.36 |
| Net effect | 1.05 | 0.74 | 1.48 | 0.80 | Net effect | 1.20 | 0.95 | 1.51 | 0.12 |

The *p*-values for the distributed lag models were adjusted for seasonality and long-term trends.

Lag-specific relative risks of recurrence and death for every 10-unit increase in annual concentrations of PM_2.5_, PM_10_, CO, or O_3_.

Lag-specific relative risks of recurrence and death for every 1-unit increase in annual concentrations of NO_2_ or SO_2_.
